# Uncovering Flavivirus Host Dependency Factors through a Genome-Wide Gain-of-Function Screen

**DOI:** 10.3390/v11010068

**Published:** 2019-01-15

**Authors:** Evgeniya Petrova, Ségolène Gracias, Guillaume Beauclair, Frédéric Tangy, Nolwenn Jouvenet

**Affiliations:** Viral Genomics and Vaccination Unit, UMR3569 CNRS, Virology department, Institut Pasteur, 75015 Paris, France; epetrova@jacobs-alumni.de (E.P.); segolene.gracias@pasteur.fr (S.G.); guillaume.beauclair@pasteur.fr (G.B.); frederic.tangy@pasteur.fr (F.T.)

**Keywords:** Flavivirus, West Nile virus, Yellow fever virus, Zika virus, Host-pathogen interactions, Virus replication, Genome-wide gain-of-function screen, Oligosaccharyltransferase complex, Ribosomal proteins

## Abstract

Flaviviruses, such as dengue (DENV), West Nile (WNV), yellow fever (YFV) and Zika (ZIKV) viruses, are mosquito-borne pathogens that present a major risk to global public health. To identify host factors that promote flavivirus replication, we performed a genome-wide gain-of-function cDNA screen for human genes that enhance the replication of flavivirus reporter particles in human cells. The screen recovered seventeen potential host proteins that promote viral replication, including the previously known dolichyl-diphosphooligosaccharide--protein glycosyltransferase non-catalytic subunit (DDOST). Using silencing approaches, we validated the role of four candidates in YFV and WNV replication: ribosomal protein L19 (RPL19), ribosomal protein S3 (RPS3), DDOST and importin 9 (IPO9). Applying a panel of virological, biochemical and microscopic methods, we validated further the role of RPL19 and DDOST as host factors required for optimal replication of YFV, WNV and ZIKV. The genome-wide gain-of-function screen is thus a valid approach to advance our understanding of flavivirus replication.

## 1. Introduction

Flaviviruses are enveloped RNA viruses that are transmitted to vertebrate hosts by mosquito or tick bites. Several members of the flavivirus genus, such as dengue virus (DENV), West Nile virus (WNV), Japanese encephalitis virus (JEV), yellow fever virus (YFV) and Zika virus (ZIKV) are highly pathogenic to humans and constitute major global health problems. Despite differences in cell tropism and pathogenesis, flaviviruses share common genomic organization, replication strategies and virion structure. Their genome consists of a single-stranded, positive-sense RNA molecule of around 10.7 kb, encoding a polyprotein precursor that give rise to seven non-structural (NS) proteins (NS1, NS2A, NS2B, NS3, NS4A, NS4B and NS5) and three structural proteins (capsid C, membrane precursor prM and envelope E) upon processing by the viral protease NS3 and its cofactor NS2B, as well as by cellular proteases [1]. The structural proteins constitute the viral particle, while NS proteins coordinate RNA replication, viral assembly and modulate innate immune responses. To promote their replication, flaviviruses rearrange membranes of the endoplasmic reticulum (ER) into novel organelle-like structures (also termed viral factories) [2].

YFV, which is the prototypic flavivirus, is responsible for viral hemorrhagic fever resulting in up to 50% fatality [3]. The virus circulates in tropical Africa and South America. The YFV vaccine 17 D is one of the most successful ever developed [4]. However, due to poor vaccine coverage and vaccine shortage, YFV regularly resurges in the African and South American continents, as illustrated by recent outbreaks, and is now emerging in Asia [5]. WNV is endemic throughout Africa, the Middle East, parts of Asia, and Europe. Since an outbreak in the USA in 1999, WNV has emerged as the most common cause of arboviral encephalitis in North and Middle America, causing millions of infections [6]. ZIKV has recently emerged in the South Pacific, South and Central Americas. Although many cases of ZIKV are asymptomatic and the most common symptoms of infection are rash, fever, and joint pain, the recent outbreaks caused alarm because they were associated with severe fetal abnormalities, including stillbirth and microcephaly, or the Guillain-Barré syndrome in infected adults [7]. ZIKV infection is now identified as a sexually-transmitted illness as well [8,9,10]. DENV, by affecting an estimated 50–100 million people per year is the most prevalent and rapidly spreading arboviral disease in humans [11]. Despite the high morbidity and mortality associated with flavivirus infections, antiviral therapies are missing. Thus, there is a pressing need to develop a deeper understanding of the interactions between flaviviruses and their cell host.

To carry out their replicative cycle, flaviviruses rely on hundreds of host gene products. Several genome-scale approaches, based on gene silencing techniques, such as siRNA-based screens [12,13,14] and more recently, CRISPR/Cas9-based screens [12,15,16,17] have been used to identify flavivirus host factors. Here, we describe a lentiviral-based gain-of-function cDNA screening method to identify candidate host factors involved in flavivirus replication.

## 2. Materials and Methods

### 2.1. Cell Lines

A549 human lung cancer cells, HeLa human cervical cancer cells, HEK293T human embryonic kidney cells, HT1080 human fibrosarcoma cells, BHK-21 baby hamster kidney cells and Vero African green monkey kidney cells were obtained from ATCC (Manassas, VT, USA). Huh7 human hepatocellular carcinoma cells were a gift from E. Meurs (Pasteur Institute, Paris, France). A549, HeLa, HEK293T and BHK-21 cell lines were cultured in Dulbecco’s Modified Eagle Medium (DMEM) (Thermo Fischer Scientific, Waltham, MA, USA) supplemented with 10% fetal bovine serum (FBS) and 1% penicillin/streptomycin (P/S) (Thermo Fischer Scientific, Waltham, MA, USA). HT1080 cells were cultured in DMEM supplemented with 5% FBS and 1% P/S. Huh7 cells were cultured in DMEM supplemented with 10% FBS, 1% P/S and 1% non-essential amino acids (Sigma-Aldrich, St. Louis, MO, USA).

### 2.2. Virus Stocks and Infection

YFV strain 17D-204 (Stamaril vaccine; Sanofi Pasteur, Lyon, France) was provided by the Pasteur Institute Medical Center. WNV Israeli strain IS-98-STI was provided by the Biological Resource Center of Pasteur Institute. ZIKV strain MR766 was provided by ATCC (Manassas, VT, USA). YFV, WNV and ZIKV virus stocks were grown in Vero cells. Viruses were concentrated by polyethylene glycol 6000 precipitation and purified by centrifugation in a discontinued gradient of sucrose. Viruses were titrated on Vero cells by plaque assay as previously described [18]. Cell infections were carried at a multiplicity of infection (MOI) of 1. The viral inoculum was replaced with fresh culture medium two hours post-infection.

### 2.3. Production and Titration of Reporter Virus Particles (RVPs)

The CMV promoter-driven WNV replicon constructs pWNVIIRep-G/Z [19] was used as a template to generate pWNVIIRep-G/Puro. The GFP-puromycin fusion cassette was generated using the Puromycin coding sequence from pQCXIP vector (Clontech Laboratories, Mountain View, CA, USA) as a template, using forward 5′-GCCCTAGATCTATGACCGAGTACAAGCCCAC-3′ and reverse 5′-GCCCTGGATCCTCAGGCACCGGGCTTGCGGGTCATGCACCA-3′ primers. The PCR product was then ligated into the BglII/BamHI sites of pEGFP-C1 vector (Clontech Laboratories, Mountain View, CA, USA). The resulting GFP-puromycin fusion construct was amplified by PCR using forward 5′-GCCCTACGCGTATGGTGAGCAAGGGC-3′ and reverse 5′-GCCCTACGCGTGGCACCGGGCTTGC-3′ primers and ligated into the MluI site of pWNVIIRep. The codon-optimized sequence the YFV structural genes was synthesized and cloned in pcDNA3.1(+) vector (Life Technologies, Carlsbad, CA, USA). RVPs were produced by transfecting HEK293T cells with pWNVIIRep-G/Puro and pcDNA3.1(+)-YFV CprME vectors at a molar ratio of 1:1 by the calcium phosphate method. One to two hours prior transfection, the cells’ medium was changed with low-glucose DMEM medium (Life Technologies, Carlsbad, CA, USA) supplemented with 10% FBS and 1% P/S. Plasmid DNA was mixed with H_2_O and 1 M CaCl_2_ solution (Sigma-Aldrich, St. Louis, MO, USA) and then an equal volume of 2x HEPES-buffered saline (pH 7.1, 140 mM NaCl (Sigma-Aldrich, St. Louis, MO, USA), 1.5 mM Na_2_HPO_4_ (Sigma-Aldrich, St. Louis, MO, USA) and 50 mM HEPES (Sigma-Aldrich, St. Louis, MO, USA) was added to the mixture drop-wise with simultaneous vortexing. The final concentration of CaCl_2_ in the transfection mix was 0.25 M. The resulting transfection mixture was incubated at room temperature for 20 min and then added to the cells. After overnight incubation, the medium of the transfected cells was replaced with fresh low-glucose medium. RVPs were harvested 48 and 72 h after transfection. The culture medium containing RVPs was centrifuged at 3000 rpm for 10 min at 4°C to remove cell debris, filtered through 0.45 μm sterile PVDF filter and stored at −80°C.

RVPs’ infectious titer was determined by infecting Huh7 cells for 48 h. The cells were fixed in 1% paraformaldehyde (PFA)-phosphate-buffered saline (PBS) solution for 10 min at room temperature, washed once with PBS and the percentage of GFP-positive cells was determined by flow cytometry. The RVPs titer (IU/mL, infectious units/mL) was calculated using the formula [(F × C)/V] × D, where F equals the frequency of GFP-positive cells (percentage obtained divided by 100), C equals the total number of cells in the well at the start of infection, V equals the volume of inoculum and D is the RVPs dilution [20].

### 2.4. cDNA Library Screen

A pooled, uncut, three-reading frame cDNA library of human A549 cells was prepared and cloned into pLenti6/DEST vector (custom-made services, Life Technologies, Carlsbad, CA, USA). A total of 1.12 × 10^7^ HT1080 cells were transduced with cDNA library lentiviruses at an MOI 0.2, resulting in two-fold representation of library complexity. Following selection with blasticidin (4 μg/mL) for 10 days, the cDNA library-transduced cells were harvested, 6.2 × 10^7^ cells (five-fold representation of cDNA library complexity) were re-seeded and, 24 h later, infected with YFV/WNV chimeric RVPs. On the following day, the cells’ medium was replaced with fresh culture medium containing blasticidin. Selection of RVPs-infected cells with puromycin (2 μg/mL) was started 48 h after infection. After selection with puromycin for three weeks, the resistant colonies (77 in total) were pooled. An aliquot (~ 5 × 10^5^ cells) was taken for genomic DNA extraction, which was done by lysing the cells in a buffer containing 100 mM NaCl, 10 mM TrisHCl, pH 8.0, 25 mM EDTA, pH 8.0, 0.5% SDS and 0.1 mg/mL proteinase K for 12 h at 50 °C, followed by phenol/chloroform extraction and ethanol precipitation. PCR for integrated cDNA clone isolation was done using forward 5′- CAGTACATCAATGGGCGTGG -3′ and reverse 5′-GGGGACTTTCCACACCCTAAC -3′ primers, 50–250 ng of genomic DNA, 1 U of Phusion DNA polymerase (Thermo Fischer Scientific, Waltham, MA, USA) and the following cycling parameters: 1× (98 °C, 30 s), 30× (98 °C, 10 s; 68°C, 20 s; 72 °C, 1 min), 1× (72 °C, 10 min; 4 °C, hold). Reactions with genomic DNA from HT1080 parental cells and HT1080 cells transduced with pLenti6-GFP were performed in parallel as negative and positive controls, respectively. NGS of PCR products from the pool of 77 clones was done at Institut Pasteur’s ‘Plateforme Transcriptome et Epigenome (PF2)’ using Illumina MiSEQ instrument. Amplification-free DNA library was prepared using NEXTflex® PCR-Free DNA Library Prep Kit (Bioo Scientific, Austin, TX, USA). The sequencing run was paired-end with a read length of 162 base pairs. Reads were mapped to the human reference genome USCS hg19 using STAR [21] with parameters adjusted to detect the junction between the lentiviral vector and the cDNA clone, thus restricting the analysis to amplicons derived from the transduced vector and excluding hits derived from possible genomic DNA contaminants. Hits with more than 10 mapped reads were considered candidate genes. For gene classification according to sub-cellular localization, DAVID database, as well as manual content curation of literature for each gene was used.

### 2.5. siRNA-Mediated Gene Silencing

siRNA oligos (Table S1) were transfected in HeLa or A549 cells (40–50% confluent) at 30 nM final concentration using Lipofectamine RNAiMax reagent (Thermo Fischer Scientific, Waltham, MA, USA) and following the manufacturer’s protocol. Transfected cells were harvested or used for infection assays 48 h after transfection.

### 2.6. Flow Cytometry Analyses

Infected cells were fixed with cytofix/cytoperm kit (BD Biosciences, Franklin Lakes, NJ, USA) and stained using the pan-flavivirus anti-Env 4G2 antibody and secondary antibodies. Non-infected, antibody-stained samples served as controls for the signal background. Data were acquired using Attune NxT Acoustic Focusing Cytometer (Life Technologies, Carlsbad, CA, USA) and analyzed using FlowJo software (version 10.1).

### 2.7. Lentiviral Production and Generation of HT1080 Stable Cell Lines

Lentiviral constructs expressing RPL19, RPS3, DDOST or TIM-1 were generated using the Gateway cloning technology (Life Technologies, Carlsbad, CA, USA). Entry clones for DDOST were obtained from Yves Jacob (Pasteur Institute, Paris, France). RPL19 and RPS3 were cloned into pDONR221 vector (Life Technologies, Carlsbad, CA, USA) using as template HEK293T or HeLa cells cDNA, respectively. TIM-1 was cloned into pDONR221 vector using pTRIP-TIM-1 vector (Ali Amara, IUH, Paris, France) as a template. The primers used for gene amplification by PCR are listed in Table S2. Stable H1080 cell lines over-expressing our selected genes were generated by lentivirus transduction. Gateway LR recombination reaction was performed to insert the cloned genes into pSCRPSY-TagRFP-DEST (Paul Bieniasz, Rockefeller University, New York, US) or pLenti-CMV-Puro-DEST (# 17452, Addgene, Cambridge, MA, USA) lentiviral vectors. pLenti6-GFP (# 35637, Addgene Cambridge, MA, USA) was used as a positive control for cDNA library lentivirus infection efficiency. Lentiviruses were produced by calcium phosphate-mediated transfection of HEK293T cells with pCMV-VSV-G codon-optimized, pCMVR8.74 (both plasmids were given by Pierre Charneau Pasteur Institute, Paris, France) and lentiviral vector at a mass ratio of 1:4:4. Lentivirus titration was done on HT1080 cells by flow cytometry (pSCRPSY-TagRFP vector) or colony formation (pLenti6 and Lenti-CMV-Puro-DEST vectors) assays. Cells stably expressing the gene of interest were selected and maintained with culture medium containing puromycin at 1 μg/mL final concentration. Cells transduced with empty vector lentivirus were used as controls.

### 2.8. RNA Purification, Reverse Transcription, and Quantitative Real-Time PCR

Total RNA was extracted from cells using NucleoSpin RNA kit (Macherey-Nagel, Düren, Germany), following the manufacturer’s protocol. cDNA was synthetized by reverse transcription of equal amounts of purified total RNA using random hexamers (Thermo Fischer Scientific, Waltham, MA, USA) and RevertAid H Minus Reverse Transcriptase (EP0451, Thermo Fischer Scientific, Waltham, MA, USA), according to the manufacturers’ instructions. Gene expression was determined by real-time qPCR using primers described in Table S3, FastStart Universal SYBR Green Master Mix (Roche, Basel, Switzerland), and a Quant Studio 6 Flex PCR instrument (Life Technologies, Carlsbad, CA, USA). Gene expression was quantified according to the ΔΔ*CT* method [22] or the ΔΔ*CT* method with correction for efficiency [23], using GAPDH as endogenous reference control. Standard curves for cellular genes were established using 10-fold serial dilutions of plasmids containing the entire cDNA of the gene of interest. The measured amounts of each mRNA were normalized to the amounts of GAPDH mRNA. The amounts of RNA were expressed as number of copies/μg total RNA. Viral genome copy number per μg RNA was determined by absolute quantification using a standard curve obtained with serial dilutions of the pYFVRneo [24], pIRES-Hyg2-WNVCprME [19] or pcDNA6.2 ZIKV [25] plasmids.

### 2.9. Immunoblot

Cells were lysed in RIPA buffer (Sigma-Aldrich, St. Louis, MO, USA) supplemented with protease and phosphatase inhibitors cocktail for 30 min at 4 °C with rotation. Lysates were cleared by centrifugation at 20,000× g and 4°C for 15 minutes. Protein extracts were mixed with LDS sample buffer (Thermo Fischer Scientific, Waltham, MA, USA) and NuPAGE reducing agent (Thermo Fischer Scientific, Waltham, MA, USA), boiled for 5 min at 95 °C and separated by SDS-PAGE on 4–12% NuPAGE bis-tris pre-cast polyacrylamide gels (Thermo Fischer Scientific, Waltham, MA, USA). Proteins were transferred onto nitrocellulose membrane (BioRad, Hercules, CA, USA) using Bio-Rad trans-blot protein transfer system. Membranes were blocked in PBS containing 0.1% Tween-20 and 5% non-fat milk for 1 h at room temperature. Membranes were subsequently incubated overnight at 4°C with primary antibodies diluted in blocking buffer. Membranes were washed with PBS containing 0.1% Tween-20 and further incubated with secondary antibodies. Membranes were scanned using Odyssey CLx imaging system (Li-COR Biosciences, Lincoln, NE, USA). The following primary antibodies were used in the study: mouse anti-RPL19 (clone K-12, sc-100830, Santa Cruz Biotechnology, Dallas, TX, USA) diluted 1:500, mouse anti-DENV NS1 17A12 [26] diluted 1:1000, anti-Env 4G2 antibody diluted 1:2000, rabbit YFV-NS4B [27] diluted 1:1000, and mouse anti-βActin (reference A5316, Sigma-Aldrich, St. Louis, MO, USA) diluted 1:5000.

### 2.10. Immunofluorescence Analysis

pDONR-223-DDOST was used as a template to clone DDOST by in vitro recombination into a Gateway compatible peGFP-N1 vector (both plasmids were provided by Yves Jacob, Institut Pasteur Paris) to generate DDOST-GFP. HeLa cells were transfected with Lipofectamine LTX (Thermo Fischer Scientific, Waltham, MA, USA) using four times less DNA and transfection reagents than recommended by the manufacturer. Following two days of transfection, cells were infected at an MOI of 1 with YFV, WNV or ZIKV. Twenty-four hours post-infection, cells were fixed with PFA 4% for 30 min at room temperature (RT). Cells were then permeabilized with PBS, 0,5% Triton X-100 for 10 min at RT, blocked with PBS containing 0,05% Tween-20, 5% BSA (PBSTA) for 30 min and stained with anti-NS1 MAb 6B8 (provided by Marie Flamand, Pasteur Institute, Paris, France) in PBSTA for one hour. Cells were then stained with secondary antibodies Alexa-Fluor 488 in PBSTA for 30 min. Nuclei were stained with NucBlue (Thermo Fischer Scientific, Waltham, MA, USA) for 15 min at RT. Images were acquired with a LSM 720 laser scanning confocal microscope equipped with an ×63 objective (Zeiss, Oberkochen, Germany).

### 2.11. Cell Viability Assays

Cell viability was determined using a trypan blue exclusion assay.

### 2.12. Statistical Analysis

Data were analyzed using GraphPad Prism 7. Statistical analysis was performed with One-way ANOVA or unpaired *t* tests. Data are presented as means ± SD of at least two independent experiments. Statistically significant differences are indicated as follows: * *p* < 0.05, ** *p* < 0.01, *** *p* < 0.001, and **** *p* < 0.0001; ns, not significant.

## 3. Results

### 3.1. Identification of Host Factors That Promote the Replication of Chimeric YFV/WNV Reporter Particles

**Figure 1:**
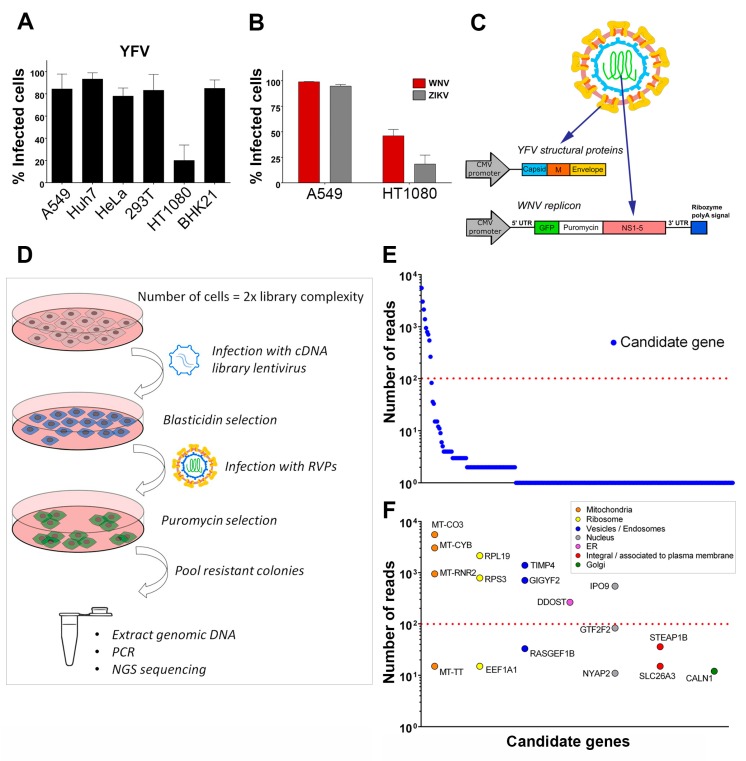
Identification of host factors that promote the replication of chimeric yellow fever (YFV)/ West Nile (WNV) reporter particles. (**A**) A panel of mammalian cell lines was infected with YFV at a multiplicity of infection (MOI) of 1 for 24 h. The percentages of cells that expressed the viral E protein were determined by flow cytometry analysis. (**B**) A549 and HT1080 cells were infected with WNV or Zika (ZIKV) at an MOI of 1. The percentages of cells that expressed the viral E protein were determined by flow cytometry analysis 24 h post-infection. (**C**) Schematic depiction of YFV/WNV chimeric reporter viral particles (RVPs). (**D**) Schematic depiction of the genome-wide gain-of-function cDNA screening approach. (**E**) Candidate genes identified by next-generation sequencing of pooled-colony genomic DNA. The Y-axis represents the number of reads that map to each gene, represented by a circle. (**F**) The genes with more than ten reads were classified according to subcellular localization. The Y-axis represents the number of reads that map to each gene. Data in (**A**) and (**B**) are represented as mean ± SD of at least three independent experiments. The red line in (**E**) and (**F**) marks a cut-off value of 100 reads per mapped gene.

To identify host factors that positively regulate flavivirus replication, we designed a strategy to screen a lentiviral cDNA library in cells in which viral replication was inefficient. We first tested the efficiency of infection of YFV, the prototypical flavivirus, in various mammalian cell lines (Figure 1A). Flow cytometric analysis revealed that four to five times more A549, Huh7, HeLa, HEK293T and BHK-21 cells were positive for YFV E protein than HT1080 fibrosarcoma cells at 24 h post-infection (Figure 1A). WNV and ZIKV replication was also less efficient in HT1080 cells than in A549 cells at 24 h post-infection (Figure 1B). A549 cells were chosen to produce the cDNA library, which was cloned into a lentiviral vector carrying a blasticidin resistance gene. To screen the library, we applied a selection method based on the use of reporter virus particles (RVPs). RVPs are pseudo-viruses that can be generated by complementation of a subgenomic replicon (encoding NS flavivirus proteins and a reporter/selection cassette) by a plasmid encoding the structural proteins (C, prM, E) provided *in trans* (Figure 1C) [19]. The RVPs-infected cells replicate the subgenomic replicon at a level that depends on the presence of host replication factors, and such cells can be selected with antibiotics. We were unable to generate YFV RVPs, possibly because YFV structural genes expressed *in trans* did not package YFV subgenomic replicon. We have previously produced RVPs carrying YFV structural proteins with the WNV replication machinery [28] and thus used these chimeric RVPs to perform the screen (Figure 1C). Since YFP and WNV replicated less efficiently in HT1080 cells than in other cells tested (Figure 1A,B), they provided suitable target cells to screen the cDNA library for genes encoding proteins enhancing viral replication. HT1080 cells were transduced with the cDNA library and selected based on antibiotic resistance (Figure 1D). Selected cells were then challenged with YFV-enveloped WNV RVPs expressing GFP and a puromycin-resistance gene (Figure 1D). Seventy-seven colonies survived the antibiotic treatment. They were pooled and an aliquot was taken for genomic DNA extraction. PCR products obtained using primers aligning to the LTR of the lentiviral vector were subjected to next-generation sequencing (NGS) analysis. Mapping of the reads onto the human genome was done with parameters adjusted to detect the junction between the lentiviral vector and the cDNA clone, thus restricting the analysis to library cDNAs and excluding hits derived from possible genomic DNA contaminants. Seventeen genes with more than ten reads were identified using this approach (Figure 1E). These genes were classified into distinct categories according to subcellular localization (Figure 1F). For further investigation, we focused on the six most enriched (> 100 reads) non-mitochondrial genes identified: The two ribosomal proteins RPS3 and RPL19, TIMP metallopeptidase inhibitor 4 (TIMP4), GRB10 interacting GYF protein 2 (GIGYF2), dolichyl-diphosphooligosaccharide--protein glycosyltransferase non-catalytic subunit (DDOST) and importin 9 (IPO9). DDOST is a part of the oligosaccharyltransferase (OST) machinery that catalyzes the N-linked glycolysation of newly-synthetized proteins in the ER [29]. Members of the OST complex, including DDOST, have been recently identified as flavivirus host-factors by different approaches, such as genome-scale CRISPR screens [12,15,16,17] and proteomic analysis of cellular factors that interact with DENV NS1 [30]. Thus, recovery of DDOST in our cDNA screen validates our strategy to identify genes promoting flavivirus replication.

### 3.2. Gene Silencing Approaches Validate the Role of Identified Candidates in YFV and WNV Replication

**Figure 2:**
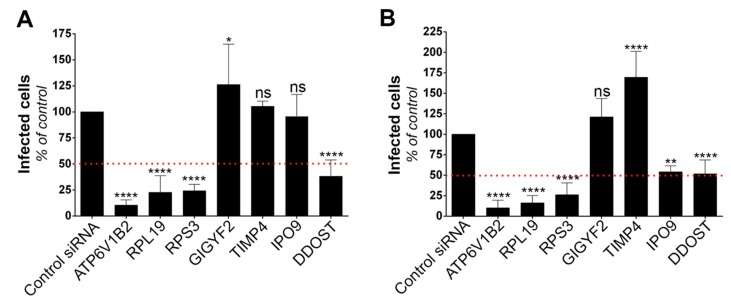
Silencing approaches validate the role of the identified genes on viral replication. Infection assays performed with YFV (**A**) and WNV (**B**) in HeLa cells 48 h following transfection with siRNA targeting candidate genes. The percentages of cells that expressed the viral E protein were determined by flow cytometry analysis 24 h post-infection. Data are normalized to non-targeting siRNA transfected cells. They are represented as mean ± SD of at least three independent experiments. Significance was calculated using one-way ANOVA tests of comparisons to control siRNA-transfected samples.

To validate the role of the six selected candidates in the context of genuine flavivirus infection, we conducted siRNA-mediated gene silencing experiments in HeLa cells, which are permissive to YFV (Figure 1A) and WNV [31]. As a positive control, we used siRNA directed against ATP6V1B2, a protein required for fusion between flaviviral and endosomal membranes [28]. Non-targeting siRNA were used as negative controls. RT-qPCR analyses confirmed that the pools of siRNA were reducing the expression of their respective targets (Figure S1). Flow cytometric analysis performed at 24 h post-infection revealed that silencing of RPL19, RPS3 and DDOST significantly impaired both YFV and WNV replication (Figure 2A,B). Reduced expression of IPO9 significantly impaired replication of WNV, but not of YFV (Figure 2A,B). The number of cells positive for the viral protein E in cells depleted of GIGYF2 or TIMP4 was either similar or higher compared to that of control cells upon YFV or WNV infection (Figure 2A,B), suggesting that these two candidates are dispensable for replication of both viruses. This does not exclude the possibility that their over-expression enhanced viral replication and thus explains their recovery through the screen.

Together, these data suggest that RPL19, RPS3 and DDOST are genuine YFV and WNV host dependency factors. To demonstrate further the usefulness of our screening approach, we focused on two of the main hit genes, DDOST and RPL19.

### 3.3. DDOST Is a Flavivirus Host Factor

**Figure 3:**
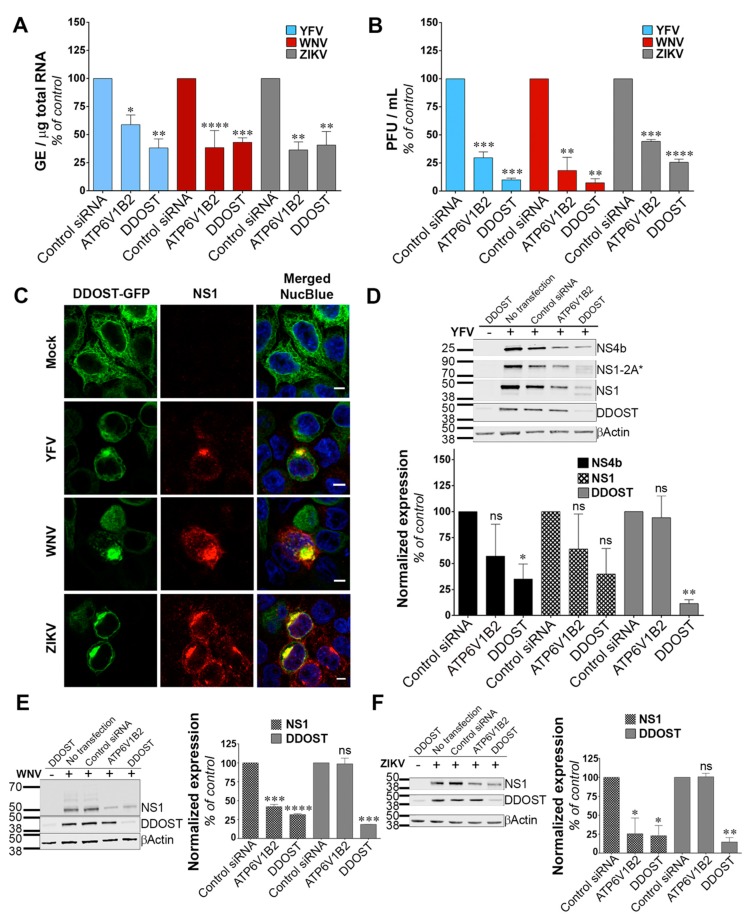
Dolichyl-diphosphooligosaccharide—protein glycosyltransferase non-catalytic subunit (DDOST) is a flavivirus host factor. (**A**) Forty-eight hours after transfection with siRNA, HeLa cells were infected with YFV, WNV or ZIKV at an MOI of 1. The relative amounts of cell-associated viral RNA were determined by qPCR analysis at 24 h post-infection. Amounts of viral RNA are expressed as genome equivalents (GE) per μg of total cellular RNA and normalized to the sample transfected with non-targeting control siRNA. (**B**) The presence of infectious virus released in the culture medium of infected HeLa cells was measured by plaque assays. Results are expressed as plaque forming units (PFU) per mL and normalized to the sample transfected with non-targeting control siRNA. (**C**) HeLa cells transiently expressing GFP-tagged DDOST were infected with YFV, WNV or ZIKV. Cells were stained with anti-NS1 (red) and NucBlue (blue) 24 h post-infection. Ten microscopic fields were analyzed Scale bars are 5 μm. (**D**–**F**) HeLa cells were infected with YFV (**D**), WNV (**E**), or ZIKV (**F**) 48 h after transfection with siRNA. The level of viral protein was determined by SDS-PAGE and Western blotting 24 h post-infection. The band signal is normalized to the loading control (βActin) and is expressed as percent of protein expression in the sample transfected with non-targeting control siRNA. Data in (**A**,**B**,**D**–**F**) are represented as mean ± SD of two independent experiments. Significance was calculated using a one-way ANOVA or unpaired t tests of comparisons to control siRNA-transfected samples.

We investigated the function of DDOST in flavivirus replication. siRNA-mediated silencing of DDOST in infected HeLa cells followed by RT-qPCR analysis revealed that DDOST expression was required for optimal YFV and WNV RNA production (Figure 3A). These data are in agreement with our previous flow cytometric analysis (Figure 2A,B). DDOST expression was also required for optimal ZIKV RNA production (Figure 3A). Furthermore, we observed a significant reduction of release of infectious YFV, WNV and ZIKV particles in the supernatant of cells that expressed a lower level of DDOST compared to control cells (Figure 3B). To investigate the localization of DDOST in HeLa cells infected with the three tested viruses, immunofluorescence microscopy on cells transiently expressing DDOST-GFP was performed. In non-infected cells, DDOST-GFP was distributed in an intracellular reticular pattern consistent with ER localization (Figure 3C). In cells infected with YFV, WNV or ZIKV, DDOST-GFP was recruited to sites of viral replication, which were identified by the presence of NS1 (Figure 3C).

Given the debate on the potential role of the OST complex for flavivirus proteins N-glycosylation [15,16,30,32], we performed immunoblot analysis for YFV-, WNV- and ZIKV-NS1, as well as for YFV-NS4B in HeLa cells silenced for DDOST. NS1 is highly conserved among flaviviruses and contains two N-glycosylation sites [33] that are important for RNA replication and pathogenesis. DENV NS4B is N-glycosylated in infected cells and NS4B of related flaviviruses are likely to be glycosylated too, since they contain two to three putative N-glycosylation sites [34]. siRNA oligonucleotides directed against the host dependency factor ATP6V1B2 [28] served as positive controls in these experiments. In YFV infected cells, antibodies against NS1 revealed the presence of NS1, but also of a polyprotein precursor consisting of NS1 and a portion of NS2A, previously called NS1-2A* (Figure 3D), [27]. In control HeLa cells, the vast majority of NS1 and NS1-2A* were detected as unique species, probably representing the fully glycosylated forms (Figure 3D). A faint NS1 band with faster mobility than the main NS1 species was sometimes observed in control cells (Figure 3D). This band likely represents a de-glycosylated form of NS1. In cells silenced for DDOST, expression of both the fully glycosylated forms of NS1 and NS1-2A* were reduced compared to control cells (Figure 3D).

Moreover, the lower NS1 species was more apparent in cells silenced for DDOST than in control cells (Figure 3D). Two lower NS1-2A* species were also observed in cells silenced for DDOST (Figure 3D). Expression of YFV-NS4B was reduced in cells silenced for DDOST compared to control cells (Figure 3D), but no lower band was observed. The effect of DDOST silencing on WNV and ZIKV NS1 proteins was similar to the one observed on YFV-NS4B, i.e., a reduced level of expression but no appearance of faster mobility band (Figure 3E,F). These data suggest that DDOST may be involved in YFV-NS1 glycosylation but not in YFV-NS4B, WNV-NS1 or ZIKV-NS1 glycosylation. Reduced expression of YFV-NS4B, WNV-NS1, and ZIKV-NS1 in cells silenced for DDOST could be caused by an indirect effect of DDOST silencing on viral replication or by a direct effect on viral protein stability. Together, these data show that DDOST is recruited to flavivirus replication sites and is required for efficient replication of YFV, WNV and ZIKV.

### 3.4. RPL19 Is a Flavivirus Host Factor

**Figure 4:**
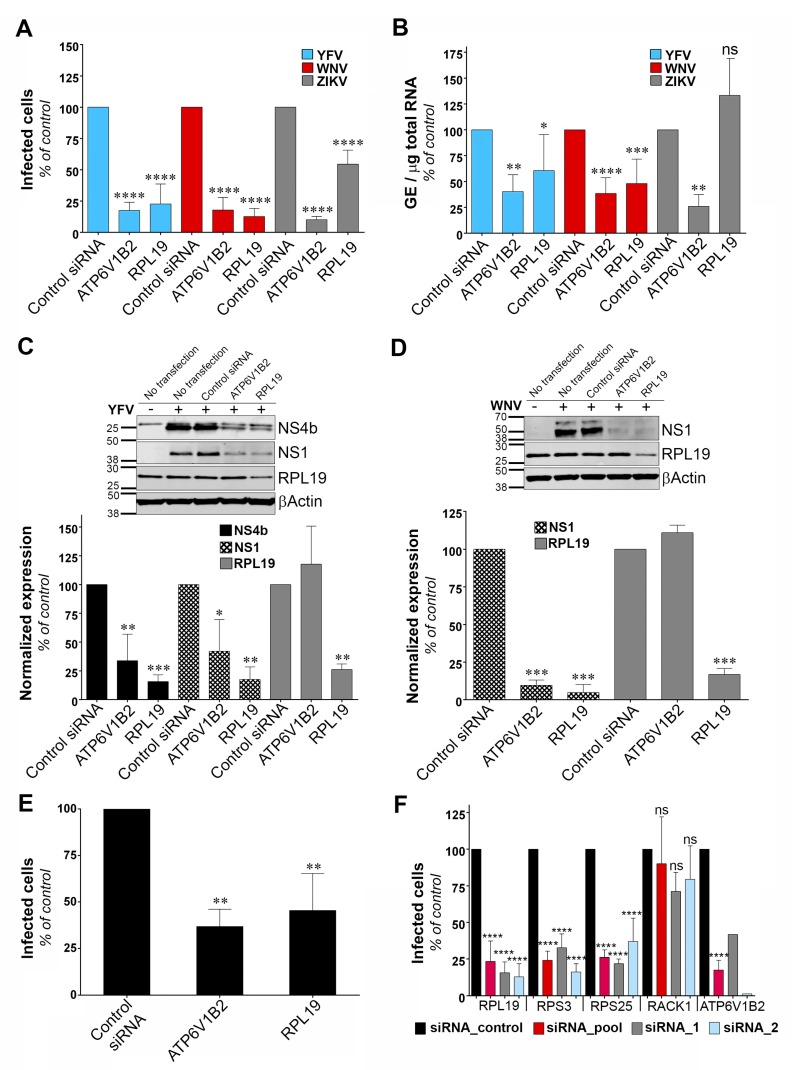
Ribosomal protein L19 (RPL19) is required for YFV and WNV viral protein synthesis. (**A**) HeLa cells were transfected with siRNA targeting RPL19 or ATP6V1B2 and infected 48 h later with YFV, WNV, or ZIKV at an MOI of 1. The percentages of cells expressing the viral E protein were determined by flow cytometry analysis 24 h post-infection. Results are expressed as percent of E-positive cells and normalized to cells transfected with non-targeting control siRNA. (**B**) HeLa cells were transfected with siRNA targeting RPL19 or ATP6V1B2 and infected with YFV, WNV, or ZIKV 48 h later at an MOI of 1. The relative amounts of cell-associated viral RNA were determined by qPCR analysis at 24 h post-infection. Amounts of viral RNA are expressed as genome equivalents (GE) per μg of total cellular RNA and normalized to the sample transfected with non-targeting control siRNA. (**C**,**D**) The level of viral protein was determined by SDS-PAGE and Western blotting 24 h after the start of infection with YFV (**C**) or WNV (**D**). The band signal is normalized to the loading control βActin) and is expressed as percent of protein expression in the sample transfected with non-targeting control siRNA. (**E**) A549 cells were transfected with siRNA targeting RPL19 or ATP6V1B2 and infected 48 h later with YFV at an MOI of 1. The percentages of cells expressing the viral E protein were determined by flow cytometry analysis 24 h post-infection. Results are expressed as percent of E-positive cells in the sample and normalized to cells transfected with non-targeting control siRNA. (**F**) HeLa cells were transfected with siRNA (pool or individual oligos) targeting RPS19, RPS3, RPS25, RACK1 or ATP6V1B2 and infected 48 h later with YFV at an MOI 1. The percentages of cells expressing the viral E protein were determined by flow cytometry analysis 24 h post-infection. Results are expressed as percent of E-positive cells and normalized to cells transfected with non-targeting control siRNA. Data are represented as mean ± SD of at least three (**A**–**C**,**E**,**F**) or two (**D**) independent experiments, at the exception of the control data obtained with individual siRNA against ATP6V1B2. Significance was calculated using a one-way ANOVA test of comparisons to control siRNA-transfected samples.

To identify the viral replication step that depends on RPL19 expression, we assessed the replication of YFV, WNV and ZIKV through viral E protein synthesis by flow cytometry in HeLa cells silenced for RPL19 (Figure 4A). siRNA oligonucleotides directed against the host dependency factor ATP6V1B2 were used as positive controls [28]. In agreement with our previous data (Figure 2A,B), knockdown of RPL19 significantly impaired viral E protein synthesis upon YFV and WNV infections, as compared to control cells (Figure 4A). The effect of RPL19 silencing on viral E protein synthesis was less pronounced upon ZIKV infection than upon YFV or WNV infection (Figure 4A). Importantly, knockdown of RPL19 did not affect HeLa cells viability (Figure S2A), confirming that the effect of RPL19 silencing on YFV and WNV replication was not caused by reduced cell proliferation. We then assessed viral RNA production by RT-qPCR in HeLa cells silenced or not for RPL19 (Figure 4B). RPL19 silencing reduced YFV and WNV RNA production around two-fold (Figure 4B). By contrast, ZIKV RNA yield was not affected by RPL19 knockdown (Figure 4B), suggesting that YFV, WNV and ZIKV differ in their dependency on RPL19 for replication. We next examined NS4B and NS1 accumulation in RPL19-depleted cells infected with YFV (Figure 4C). Quantitative Western blot analysis revealed a reduction of around 80% of NS4B and NS1 expression in cells depleted of RPL19, compared to control cells (Figure 4C). A similar reduction of WNV NS1 (around 90%) was observed upon RPL19 depletion in WNV infected cells (Figure 4D). RPL19 expression was also required for optimal YFV E production in A549 cells (Figure 4E), without affecting their viability (Figure S2B). Together, these data show that RPL19 depletion had a stronger effect on viral translation (Figure 4A,C–E) than on viral RNA production (Figure 4B), suggesting that RPL19 is primarily required for efficient YFV and WNV protein production. One can envisage that a primary effect on NS protein production triggers a secondary effect on the level of viral RNA synthesis. RT-qPCR analysis performed on cells silenced for RPL19 expression and infected for six hours with YFV showed that reduced expression of RPL19 had no significant effect on early step of viral replication (Figure S3), supporting an effect of RPL19 on viral translation rather than viral RNA production or viral entry.

To validate further the role of RPL19 in viral replication, stable HT1080 cell lines over-expressing RPL19 were generated. HT1080 cell lines over-expressing DDOST were used as a positive control. HT1080 cells over-expressing TIM-1, a protein known to enhance YFV entry into mammalian cell [35], as well as HT1080 cells over-expressing RPS3, were also included in the analysis. RT-qPCR analysis confirmed that transduced cells expressed more RPL19, RPS3, DDOST and TIM-1 than control cells transduced with an empty vector (Figure S4A). To analyze the effect of over-expressing these proteins in an experimental set-up that resembles the one used during the cDNA library screening, cells were challenged with the YFV/WNV chimeric RVPs expressing GFP (Figure S4B). Of note, the over-expression of the cDNAs used during the screen was driven by a CMV promoter whereas these experiments were conducted with a lentiviral vector containing an LTR promoter. RVPs make only a single round of infection [19]. Fluorescence intensity of GFP positive cells was measured to assess viral replication. As expected from our previous data (Figure 3), cells over-expressing DDOST produced about four- to five-fold more GFP than control cells (Figure S4B). Cells over-expressing RPL19 and RPS3 produced about two-fold more GFP than control cells (Figure S4B), albeit this increase was not statistically significant. As expected for a viral entry factor, TIM-1 over-expression did not enhance viral replication in a given infected cell (Figure S4B). These data suggest that over-expressing RPL19 or RPS3 favors the replication of the WNV-GFP replicon and probably not a viral entry step.

To determine whether ribosomal proteins other than RPL19 and RPS3 are necessary for YFV replication, we silenced RPS25 and RACK1, two components of the 40S subunit of the ribosome that were recently identified as host factors for DENV [30]. In agreement with our previous data (Figure 2A), knockdown of RPL19 and RPS3 caused a significant reduction in the percentage of cells positive for the viral E protein (Figure 4F). Similarly, knockdown of RPS25 impaired YFV replication (Figure 4F). By contrast, production of YFV E protein was not affected by RACK1 silencing (Figure 4F). RT-qPCR analysis showed that gene knockdown with two independent siRNAs or with a siRNA pool resulted in around 80% reduction of expression of each target genes in HeLa cells (Figure S5A). Cell viability was not affected by silencing these four proteins individually (Figure S5B). Together, these data suggest that YFV replication is dependent on a subset of ribosomal proteins.

## 4. Discussion

All the large-scale genetic perturbation screens that have been performed so far in the context of flaviviral infection consisted of loss-of-function screens based on either siRNA or CRISPR approaches [12,13,14,15,16,17]. Limitations of these loss-of-function screens include lack of sensitivity due to incomplete knockdown efficiency, false-negative results because of certain siRNA or gRNAs lacking activity and false-positive candidate genes due to potential off-target effects. We performed a genome-wide gain-of-function screen by over-expressing into HT1080 cells a library of cDNA generated from A459 cells. This approach, based on the generation of RVPs expressing antibiotic selection cassettes, can be adapted to any flavivirus. Our screen allowed the recovery of 17 potential flavivirus host dependency factors, including the previously known factor DDOST. The role of four candidates in YFV and WNV replication (RPL19, RPS3, DDOST and IPO9) was validated using siRNA-silencing assays, demonstrating the utility and relevance of our lentiviral overexpression screening strategy. However, as with any other genetic screens, our approach has its limitations, including the identification of false positive genes. GIGYF2 may be one of them since its reduced expression did not perturb WNV replication. GIGYF2 could have been selected in the screen because its integration in the genome of HT1080 cells disrupted the expression of a gene with antiviral properties. False positive genes may also arise during amplification by PCR of genomic DNA that was not the result of lentiviral integrative events. To limit this, we discarded non-integrated genes from the analysis. Another caveat of genome-wide gain-of-function screens is the potential loss of ORFs during the generation of the cDNA library, especially long ones. In sum, exploiting both gain- and loss-of-function screening strategies in the context of flavivirus infection should provide complementary information.

Two of our main hits were the ribosomal proteins RPL19 and RPS3. The screen also recovered EEF1A1, a ribosome associated translation elongation factor. We showed that RPL19 is required for efficient YFV and WNV protein production. ZIKV seemed less dependent on RPL19 than YFV and WNV for efficient replication. Other ribosomal proteins, such as RPS25, RPL18, RPL7, RPL1 and RPLP2 were previously recovered in genome-wide loss-of-function screens performed in the context of DENV, YFV and WNV infections [13,15,36]. Moreover, several ribosomal proteins, including RPS3 and RPL19, were identified as DENV NS1 binding partners using affinity chromatography and immunoprecipitation assays, both in cells over-expressing NS1 [37] or replicating a DENV replicon [30]. Among the dozen ribosomal proteins identified as DENV NS1 partners, RPL18 and RPS25 were chosen for further investigations and shown to be required for DENV protein translation [30,37]. Similarly, knockdown of the ribosomal proteins RPLP1/2 strongly reduced DENV protein accumulation, suggesting a requirement for RPLP1/2 in viral protein translation [36]. These data, together with ours, suggest that flaviviruses depend on a subset of ribosomal proteins for efficient replication. One can wonder why, among the 80 ribosomal proteins that exist, only a small subset was recovered in our screen and others [13,15,36]. RPL19 and RPS3 may have been the most abundant ribosomal proteins in the cDNA library that we generated. Alternatively, as suggested [30,37], viral NS proteins may recruit and/or anchor ribosomes to the ER via direct (or indirect) interaction with a subset of ribosomal proteins, including RPL19 and RPS3. Finally, one could also envisage that RPL19 and RPS3 have pro-viral functions that are not linked to their ribosomal activities [38]. Indeed, RPL19 has been proposed to inhibit IRF3 activation in HEK293 cells over-expressing TLR3 [39]. However, despite our efforts, we were unable to establish a link between RPL19 expression and antiviral IRF3-mediated interferon signaling.

DDOST was among the top-ranked hits of our screen. The human OST complex, which catalyzes the transfer of a preassembled oligosaccharide to selected asparagine residues of polypeptide chains, consists of seven subunits: Ribophorin I (RPN1), ribophorin II (RPN2), DDOST/OST48, DAD1, STT3 (isoform A or B), OST4 and N33/Tusc3 or IAP/MAGT1 [40]. Our results showed that DDOST is required for efficient replication of YFV, WNV and ZIKV in HeLa cells. This is in line with data showing that STT3A and OSTC expression is required for ZIKV, JEV, DENV and YFV replication in HEK293T cells [17], as well as with data showing the dependency of DENV, YFV, WNV and ZIKV replication on STT3B in HAP1 cells [15]. However, surprisingly, replication of YFV and WNV was not dependent on STT3A or STT3B in Huh7.5.1 cells [16]. Together, these results suggest that different flaviviruses rely on different OST subunits for replication. The use of different cell types may also account for some of the discrepancies described for flavivirus OST-dependency. Immunofluorescence and electron microscopy analysis have revealed co-localization of HA-tagged MAGT1 and STT3B with DENV replication compartments [15,16], which is in line with our experiments showing recruitment of DDOST-GFP at viral replication sites during YFV, WNV and ZIKV infection. Direct interactions between STT3A- and DENV-NS2B and -NS3 proteins have also been documented in the context of viral infection [15] and between DDOST and ectopically expressed DENV-NS1, -NS3 and -NS4B [30]. Glycosylation of the flaviviral proteins M, E, NS1 and NS4B is key for viral replication and pathogenesis, as well as for viral dissemination in mammalian hosts and mosquito vectors [34,41,42,43]. Whether the OST complex favors flavivirus replication in a glycosylation-dependent or -independent manner is still a matter of debate. Since DENV replication is drastically inhibited in OST-silenced cells [15,16], the assessment of viral protein expression and glycosylation were performed in cells exogenously expressing DENV NS1 and NS4B [16,30]. These experiments concluded that the OST complex participates in DENV NS1 and NS4B glycosylation. Our Western blot analysis, which were performed in cells infected with YFV, WNV and ZIKV and silenced for DDOST expression, suggest that DDOST could be involved, at least in part, in YFV-NS1 glycosylation, but not in YFV-NS4B, WNV-NS1 or ZIKV-NS1 glycosylation. However, reduced expression of YFV-NS4B, WNV-NS1, and ZIKV-NS1 in cells silenced for DDOST was observed, suggesting an effect of the OST machinery on the stability of these NS proteins. We propose that the OST complex acts on flavivirus replication by glycosylating a specific subset of viral proteins, such as YFV-NS1, but also by non-canonical functions that stabilize NS proteins, such as YFV-NS4B, WNV-NS1, and ZIKV-NS1. Glycosylation-independent activities of the OST complex may include structural scaffolding function [15], the catalytic oxidoreductase activity of MAGT1 [16] and/or promotion of viral protein stability. Alternatively, the OST complex could also favor flavivirus replication via the glycosylation of a proviral cellular factor.

Four mitochondrial genes were identified during the screen (MT-CO3, MT-CYB, MT-RNR2 and MT-TT). It will be of interest to investigate further their potential role in flavivirus replication. A recent proteomic analysis identified a dozen mitochondrial proteins as partners of DENV replication complexes [30]. Moreover, DENV infection disrupts mitochondria-associated membranes (MAMs), which are ER-connected interfaces critical for innate immune signaling [2,44]. Manipulation of specific mitochondrial proteins or MAMs by flaviviruses may thus create a favorable replicative environment by protecting flaviviruses from innate immunity and/or by promoting cell survival.

Another main hit from the screen is TIMP4. However, silencing its expression had no effect on YFV and WNV replication in Hela cells, suggesting that it is dispensable for efficient replication of these two flaviviruses. TIMP4-mediated enhancement of viral replication maybe cell-type specific. Of note, TIMP4 was identified in a yeast-hybrid screen as a partner of the protein 2A of the enterovirus coxsackievirus B3, a positive-strand RNA virus unrelated to flaviviruses [45] and may be involved in coxsackievirus pathogenesis [46].

Finally, our data showed that IPO9, which functions mainly in nuclear protein import, was necessary for optimal WNV replication but not required for YFV replication. Its precise role in viral replication, as well as its specificity towards WNV remains to be explored.

## 5. Conclusions

Our genome-wide gain-of-function screening strategy is a valid approach to identify flavivirus host dependency factors. It advances our understanding of flavivirus replication and provides directions for future detailed functional analysis of identified genes.

## Supplementary Materials

The following are available online at http://www.mdpi.com/1999-4915/11/1/68/s1. Figure S1: Validation of siRNA-mediated gene silencing. Figure S2: Cell viability determination following siRNA-mediated RPL19 silencing. Figure S3: Silencing of RPL19 does not affect early stages of YFV replication. Figure S4: Cell viability determination following siRNA-mediated ribosomal gene silencing and validation of gene silencing efficiency. Figure S5: Cell viability determination following siRNA-mediated ribosomal gene silencing and validation of gene silencing efficiency. Table S1: siRNA oligos used for gene silencing experiments. Table S2: DNA sequence of primers used for real-time qPCR.
