# Supplementary material for: Uncovering Flavivirus Host Dependency Factors through a Genome-Wide Gain-of-Function Screen
Source: Viruses. 2019 Jan 15;11(1):68. doi: 10.3390/v11010068 (PMC6356745; doi:10.3390/v11010068)
Supplement: Supplementary file 1 [file viruses-11-00068-s001.pdf]

Supplementary Materials

Uncovering flavivirus host dependency factors through a genome-wide gain-of-function screen

Evgeniya Petrova, Ségolène Gracias, Guillaume Beauclair, Frédéric Tangy, and Nolwenn Jouvenet

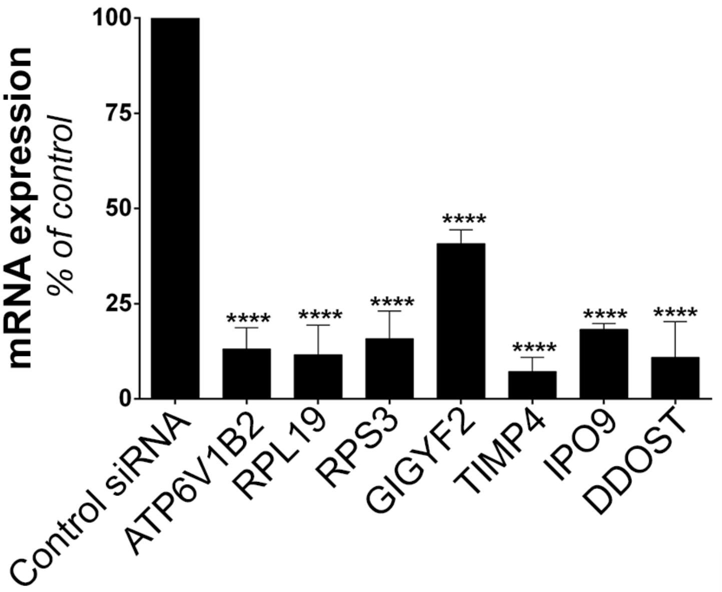

Fig. S1. Validation of siRNA-mediated gene silencing. Validation of siRNA-mediated gene silencing by RT-qPCR 48 hours after siRNA transfection of HeLa cells. Data are represented as mean ± SD of three independent experiments. Significance was calculated using a one-way ANOVA test of comparisons to control siRNA-transfected samples.

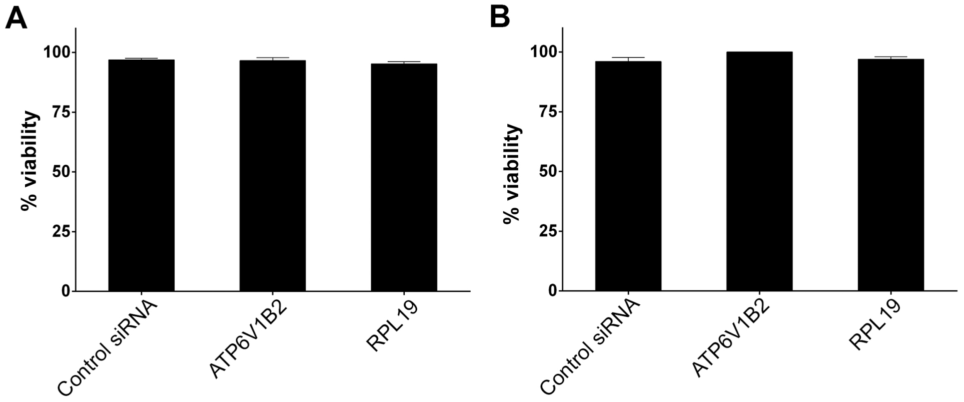

Fig. S2. Cell viability determination following siRNA-mediated RPL19 silencing. Percent viability of HeLa (A) or A549 (B) cells determined by trypan blue exclusion assays 48 hours after transfection with siRNA. Data are represented as mean ± SD of three independent experiments.

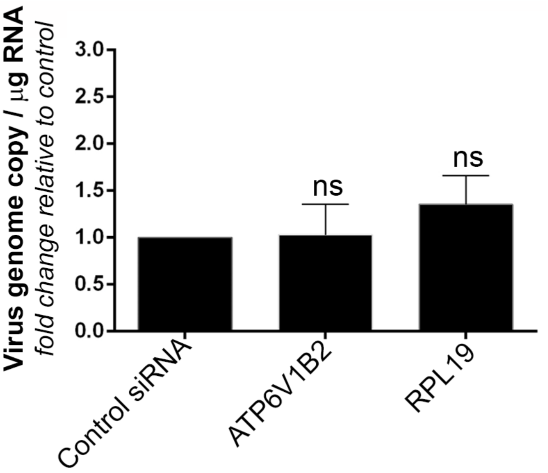

Fig. S3. Silencing of RPL19 does not affect early stages of YFV replication. HeLa cells were transfected with siRNA targeting RPL19 or ATP6V1B2 and 48 hours later infected with YFV at a MOI of 1. The relative amounts of cell-associated viral RNA were determined by qPCR analysis at 6 hours post-infection. Amounts of viral RNA are expressed as genome equivalents (GE) per μg of total RNA. Data are represented as mean ± SD of three independent experiments. Significance was calculated using a one-way ANOVA test of comparisons to control siRNA-transfected samples.

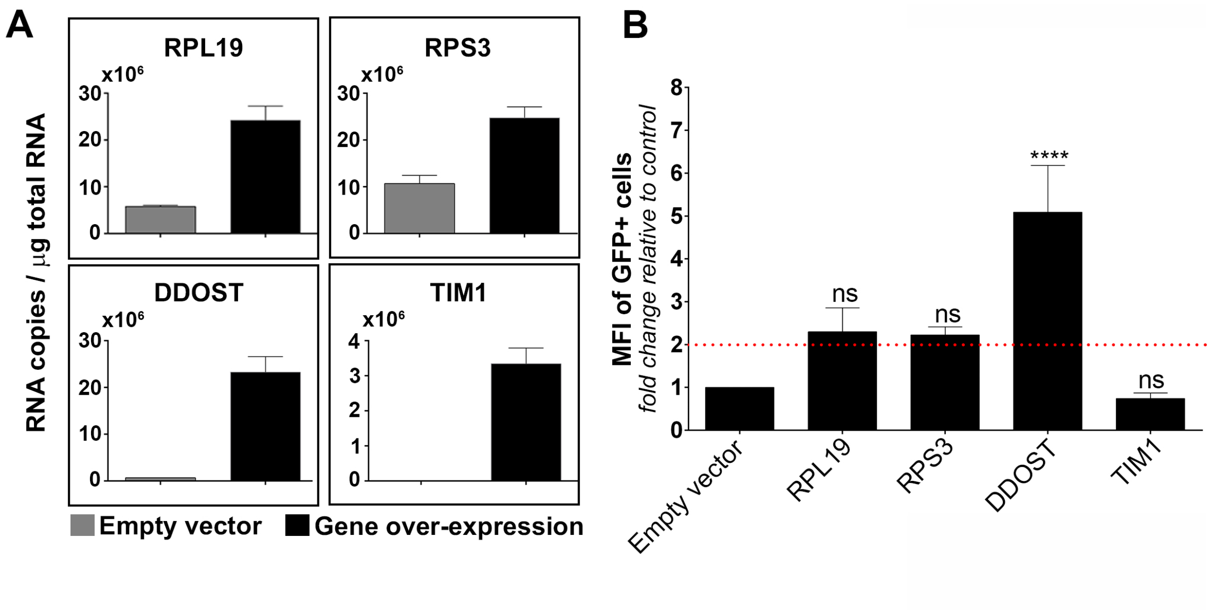

Fig. S4. Validating the role of RPL19 on viral replication by an over-expression approach. (A) Validation of gene over-expression in HT1080 stable cell lines by RT-qPCR. The results are expressed in RNA copies/µg of total RNA and are represented as mean ± SD of three independent experiments. (B) Replicon assays were performed with YFV/WNV chimeric RVPs in HT1080 cell lines over-expressing RPL19, RPS3, DDOST or TIM-1. Median Fluorescence Intensity (MFI) of GFP positive cells were determined by flow cytometry analysis 48 hours post-infection and normalized to cells transduced with an empty vector. Data are represented as mean ± SD of at least three independent experiments. Significance was calculated using one-way ANOVA tests of comparisons to empty vector cell line.

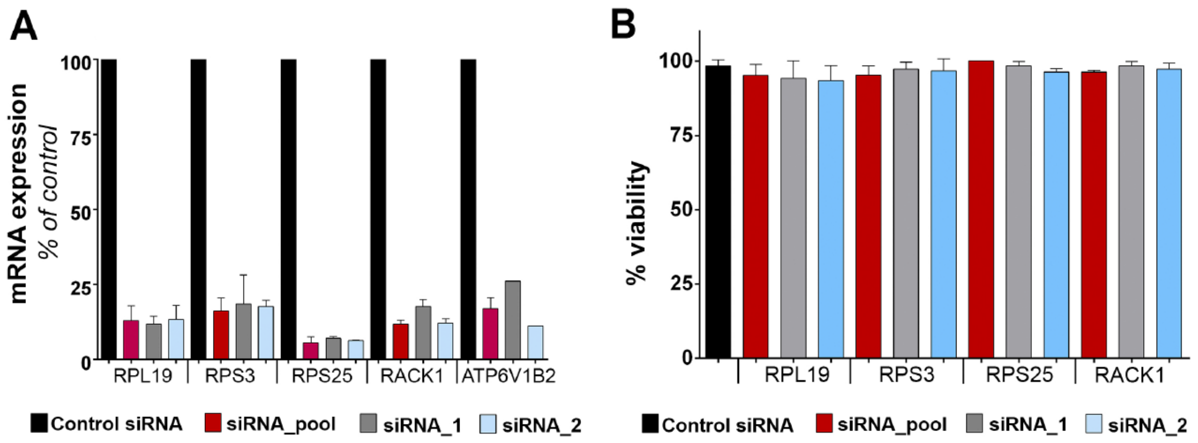

Fig. S5. Cell viability determination following siRNA-mediated ribosomal gene silencing and validation of gene silencing efficiency. (A) Comparison of gene silencing efficiency between two different siRNA oligos and a pool of siRNA oligos in HeLa cells. mRNA expression level of silenced genes was determined by RT-qPCR 48 hours post-transfection. (B) Percent viability of HeLa cells determined by trypan blue exclusion assay 48 hours after transfection with siRNA.Data are represented as mean ± SD of three independent experiments.

Target	Provider	siRNA type, reference number
Non-targeting control	ThermoFischerScientific	Silencer® Select Negative control No.1 siRNA, 4390843
	Dharmacon	ON-TARGETplus SMARTpool, D-001810-10
GAPDH	Dharmacon	ON-TARGETplus SMARTpool, D-001830-10
RPL19	ThermoFischerScientific	Silencer® Select Pre-Designed siRNA, ID: s12183
		Silencer® Select Pre-Designed siRNA, ID: s226957
RPS3	ThermoFischerScientific	Silencer® Select Pre-Designed siRNA, ID: s12255
		Silencer® Select Pre-Designed siRNA, ID: s12256
GIGYF2	ThermoFischerScientific	Silencer® Select Pre-Designed siRNA, ID: s25032
		Silencer® Select Pre-Designed siRNA, ID: s25033
		Silencer® Select Pre-Designed siRNA, ID: s25034
	Dharmacon	ON-TARGETplus SMARTpool, L-013918-01
DDOST	ThermoFischerScientific	Silencer® Select Pre-Designed siRNA, ID: 3999
	Dharmacon	ON-TARGETplus SMARTpool, L-015786-01
TIMP4	Dharmacon	ON-TARGETplus SMARTpool, L-011406-00
IPO9	ThermoFischerScientific	Silencer® Select Pre-Designed siRNA, ID: 31300
ATP6V1B2	ThermoFischerScientific	Silencer® Select Pre-Designed siRNA, ID: 7178
		Silencer® Select Pre-Designed siRNA, ID: 112786
RPS25	ThermoFischerScientific	Silencer® Select Pre-Designed siRNA, ID: s12336
		Silencer® Select Pre-Designed siRNA, ID: s53506
RACK1	ThermoFischerScientific	Silencer® Select Pre-Designed siRNA, ID: s20340
		Silencer® Select Pre-Designed siRNA, ID: s20341

Table S1. siRNA oligos used for gene silencing experiments.

Target	Primer	**DNA sequence, 5'- 3'**<br>attB1/attB2 sequence, *gene-specific sequence*
RPL19	forward	GGGGACAAGTTTGTACAAAAAAGCAGGCTTCACC*ATGAGTATGCTCAGGCTTCAG*
	reverse	GGGGACCACTTTGTACAAGAAAGCTGGGTTTTA*TTATTTCTTGGTCTCTTCCTCCT*
RPS3	forward	GGGGACAAGTTTGTACAAAAAAGCAGGCTTCACC *ATGGCAGTGCAAATATCCAAGAAG*
	reverse	GGGGACCACTTTGTACAAGAAAGCTGGGTTTTA*TTATGCTGTGGGGACTGGCT*
TIM1	forward	GGGGACAAGTTTGTACAAAAAAGCAGGCTTCACC *ATGCATCCTCAAGTGGTC*
	reverse	GGGGACCACTTTGTACAAGAAAGCTGGGTTTTA*TTAGTCCGTGGCATAAAGACTATTCTC*

Table S2. DNA sequence of primers used for cloning.

Target	Primer	DNA sequence, 5’- 3’	Reference
YFV NS3	forward	GCGTAAGGCTGGAAAGAGTG	-
	reverse	CTTCCTCCCTTCATCCACAA	
WNV Capsid	forward	CCGCGTGTTGTCCTTGATT	Fernandez-Garcia et al., 2010
	reverse	GCCCCTTGCCGTCGAT	
ZIKV NS5	forward	AARTACACATACCARAACAAAGTGGT	Faye et al., 2013
	reverse	TCCRCTCCCYCTYTGGTCTTG	
GAPDH	forward	GGTCGGAGTCAACGGATTTG	-
	reverse	ACTCCACGACGTACTCAGCG	
RPL19	forward	GGGCATAGGTAAGCGGAAGG	Bee et al., 2011
	reverse	TCAGGTACAGGCTGTGATACA	
RPS3	forward	GCGAGTTACACCAACCAGGA	-
	reverse	CCCTCTGGAAAGCCAAACCT	
GIGYF2	forward	ACGCAGACACTGAACTTTGG	-
	reverse	CATTTCTTCTCTGCCGTAACGA	
DDOST	forward	CCGACATTGGTGACCCTCTTC	-
	reverse	TCAGCCACGATGAGCGTATG	
TIMP4	forward	AACTGTGGCTGCCAAATCAC	-
	reverse	GCTTTCGTTCCAACAGCCAG	
ATP6V1B2	forward	GAGGGGCAGATCTATGTGGA	Coffey et al., 2014
	reverse	GCATGATCCTTCCTGGTCAT	
RPS25	forward	GGACGACAAGAAGAAGAAGGAC	-
	reverse	GAACTTTGCCTTTGGACCACT	
RACK1	forward	TAACCGCTACTGGCTGTGTG	-
	reverse	GTTCTGCCTTGCTGCTGGTA	
IPO9	forward	AGATGTTGGTGAGCGGAGAC	-
	reverse	CTGGGAGAATGACAGGAGCA	
TIM1	forward	CCAGTAGCCACTTCACCATCTTCAC	Zhao et al, 2010
	reverse	CGGTGTCATTCCCATCTGTTGTG	

Table S3. DNA sequence of primers used for real-time qPCR.
